# Effect of the Concentration, pH, and Ca^2+^ Ions on the Rheological Properties of Concentrate Proteins from Quinoa, Lentil, and Black Bean

**DOI:** 10.3390/foods11193116

**Published:** 2022-10-06

**Authors:** Julián Quintero, Juan D. Torres, Ligia Luz Corrales-Garcia, Gelmy Ciro, Efren Delgado, John Rojas

**Affiliations:** 1 College of Pharmaceutical and Food Sciences, University of Antioquia, Calle 67 No. 53-108, University Campus, Medellín 050010, Colombia; 2 Facultad de Ciencias y Biotecnología, Universidad CES, Calle 10 # 22-04, Medellín 050018, Colombia; 3 Department of Family and Consumer Sciences, College of Agriculture, Consumer and Environmental Sciences, New Mexico State University, NMSU Gerald Thomas Hall Room, 308 P.O. Box 30003 MSC 3470, Las Cruces, NM 88003, USA

**Keywords:** plant proteins, rheological behavior, techno-functional properties

## Abstract

Given consumer trends propelling a movement toward using plant protein in the food industry and searching for alternative protein ingredients by the industry, this study aimed to assess the influence of factors such as protein concentration, medium pH, and the presence of a divalent ion (Ca^2+^) upon the rheological properties such as viscosity change and gel formation of dispersion proteins extracted from quinoa, black beans, and lentils. A solution of each protein was prepared by varying its concentration (2.5%, 5.0%, and 10%), the pH (5.0, 7.0, and 9.0), and the incorporation of calcium chloride (0.0% and 1.0%). Each obtained solution was subjected to rheological tests to determine the parameters: consistency index (K), flow behavior (n), the storage (G’) and loss (G’’) modules, and the phase shift angle (δ). The results demonstrate that the incorporation of Ca^2+^, the shift in protein levels, and the decrease in pH modified the rheological behaviors of proteins, which were also influenced by the structural characteristics of each protein studied. However, thermal treatment and protein concentrations caused the most significant impact on proteins’ rheological behavior, forming gels independently of other conditions. It was possible to study and interpret the studied proteins’ rheological variations according to the environment’s conditions.

## 1. Introduction

Currently, the food industry requires ingredients for developing healthy products aligned with consumer trends, and over the last few years, the demand for protein ingredients has risen. The global protein ingredient market was valued at United States Dollar (USD) 38 billion in 2019 and is expected to grow by 9.1% from 2020 to 2027 [1]. Protein consumption has increased considerably, with animal proteins being the main sources so far, such as those obtained from livestock (bovine serum or beef meat), poultry (egg protein), fish, and dairy (whey) [2]. The animal protein market will continue to grow because whey and other animal proteins are essential in some formulations of diet supplements and processed food.

However, the animal protein market’s future growth may not be as accelerated due to its high production cost, its need for extensive cropping areas, and its inefficient transformation process from vegetable to animal protein (6 kg of plant protein produces 1 kg of raw animal protein), generating a strong environmental impact [3].

The increase in people adopting a vegetarian or vegan lifestyle and consumers’ desire to acquire purchasing habits that can improve the environment are gaining prominence, propelling a movement toward using plant protein in the food industry and a search for alternative protein ingredients [2]. The market for plant protein and alternative protein ingredients is expected to grow significantly because these proteins can be produced at competitive prices and other non-allergenic proteins can be obtained (since eggs, dairy, soy, gluten, and peanuts are among the “big eight” significant allergens recognized by the Food and Drug Administration) [1]. Plant proteins are currently widely utilized in the food industry due to their potential functional characteristics; such as foam formation, emulsification, gelation, solubility, film-forming, and the interaction that some vegetable proteins present with molecules of high use in the industry, such as starch. As is the case of protein from mung bean, the intersection of protein–starch is an interaction that enhances the retention capacity of the water molecules in the system, improves the system’s clarity, reduces the syneresis of gels, and extends the useful life of the products [4].

Some plant sources, such as quinoa, beans, peas, chickpeas, oats, corn, rice, and sunflower seeds, are remarkable for their high protein content ranging from 18% to 32% [5,6,7,8]. These plant protein sources also provide essential amino acids and, due to structural modifications, could serve as bioactive peptides [9,10,11,12]. Additionally, plant proteins could be used in different food production processes because of their functional properties, such as their water and oil retention, foaming capacity, viscosity, and use as a gelling agent and even as carrier material for the microencapsulation process of bioactive compounds [2,13]. However, one of the main challenges for the use of vegetable proteins in the food industry is to modify them or identify the conditions of the environment so that they can match or exceed the solubility capabilities in wide ranges of pH, gel formation, viscous capacity, digestibility, and nutritional quality that animal proteins have today.

Quinoa (*Chenopodium quinoa*), lentils (*Lens culinaris*), and black bean bushes (*Phaseolus vulgaris*) are protein-rich plant sources. Quinoa is a pseudo-cereal mainly cultivated in the Andes of South America. It contains between 12% and 20% protein, primarily globulins and albumins (Mw 8–39 kDa). Quinoa has essential amino acids, such as lysine, absent in other grains and wheat [5]. Lentils have a protein content between 24% and 30%, depending on the genotype and growing conditions [6,7], and their proteins are composed of globulins and albumins, with a molecular weight ranging from 14 to 66 kDa. Some studies have reported that proteins isolated from lentils have good emulsifying and water and oil retention ability [14,15]. Alternatively, the black bean has a protein content ranging from 20% to 30% *w/v* and exhibits a low content of sulfur-type amino acids. The globulin type extracted from black beans has a molecular weight ranging from 22 to 186 kDa. These globulins have been used to stabilize emulsions and as foaming agents [8].

In previous work, extraction process parameters were optimized to obtain quinoa, black bean, and lentil protein isolates [16]. They demonstrated strong gelling capacity, emulsifier capacity, and water and oil adsorption capacity comparable to soybean protein (data unpublished). This functionality may provide an opportunity for these proteins to be used for both food and non-food applications. The possibility of proteins to modify the medium’s viscosity and form gels is based on (i) the ability to self-associate, (ii) interactions of the hydrophobic and hydrophilic regions in the protein’s structure with the medium, (iii) the amphiphilic character, and (iv) the flexibility in their chains [17]. Globular proteins in aqueous solutions have a folded structure where the hydrophobic regions are inside a compact sphere-like structure, and the hydrophilic regions located outside the structure prompt interaction with the medium. Alternatively, the protein’s denaturation, treatments such as heating, ionic strength modification of the solvent, sonication, or pH changes, eases peptide chain mobility. Consequently, new hydrophobic interactions, hydrogen bonds, electrostatic interactions, and covalent disulfide bonds are generated, leading to changes in solvent viscosity and the formation of gels with different morphologies [18]. This study aims to elucidate the influence of factors such as protein concentration, medium pH, and the presence of a divalent ion (Ca^2+^) on the rheological properties of the dispersion proteins extracted from quinoa, black beans, and lentils. The correlation of rheological properties and the effects of process conditions on protein’s functional properties, such as viscosity change and gel formation, will potentiate the use of proteins from plant sources in the food industry.

## 2. Materials and Methods

### 2.1. Materials

Quinoa, black bean, and lentil seeds were obtained from a farmer’s market in Medellín, Colombia. Fresh seeds were oven-dried (IMP180, Thermo Fisher Scientific, Waltham, Massachusetts, United States) at 37 °C for 48 h, followed by milling. The fraction that passes through a #60 mesh sieve (<250 μm) was employed and stored in desiccators until further use. Sodium hydroxide (NaOH) (Lot B0312298904), calcium chloride (CaCl_2_) (Lot 338TA473682), tris (hydroxymethyl) aminomethane (Lot 8382C011933), concentrated hydrochloric acid (Lot SHBJ6587), and ammonium sulfate (Lot 311A675017) were obtained from Merck (Darmstadt, Germany). Sulfuric acid (Lot V01C11) was purchased from J.T. Baker (Milan, Italy).

### 2.2. Extraction of Protein Concentrates

Proteins isolated from quinoa, black bean, and lentil flours were obtained using ultrasound-assisted extraction in an alkaline medium, as previously described [16,19]. Briefly, dry powders from each source were mixed with 0.1 M tris-HCl buffer adjusted to pH 10. The buffer-to-material ratio was 5:1 for quinoa and black bean and 10:1 for lentils. Samples were sonicated (E30 h, Elmasonic) at 37 kHz, and a power of 320 W was applied for 20 min. Subsequently, samples were stirred for 1 h and filtered. The supernatant was saturated with 100% ammonium sulfate and centrifuged (Z206A, Hermle) at 4000 *g* for 30 min. Once the proteins were separated, the supernatant was discarded, and sediment was dialyzed using 3 kDa cellulose membranes (Fisherbrand™) for two days under cooling using deionization water. The obtained proteins were freeze-dried and stored in desiccators until use. Protein content was assessed according to the Association of Official Analytical Chemists methods (Association of Official Agricultural Chemists (A.O.A.C. 2011.04) [20], resulting in 35.0 ± 2.03%, 65.0 ± 1.87%, and 41.4 ± 2.98% (nitrogen content × 5.3) for quinoa, black beans, and lentils, respectively.

### 2.3. Protein Characterization

The proteins extracted under the conditions described previously were characterized by relevant parameters such as free thiol group (SH) content, molecular weight (Mw), isoelectric point (pI), and amino acid composition according to methodologies described in previous studies [16,19]. 

### 2.4. Rheological Studies

The effect of quinoa, black bean, and lentil protein solutions in different concentrations over their rheological properties was evaluated by flow curves, frequency sweeps, and temperature sweeps [11]. Those tests allowed for studying the viscoelastic behavior and gel-forming ability of plant protein [11]. Furthermore, flow curves and frequency sweeps were performed before and after each thermal treatment (80 °C during 5 min in the rheometer) to identify rheological changes. An Anton Paar brand MCR 92 rheometer (Graz, Austria) was used for evaluating these rheological tests with Rheocompass^®^ software (v.1.20, Anton Paar) and a C-CC27 concentric cylinder geometry (27 mm diameter). The protein concentrate concentration effect on rheological parameters was evaluated using solutions for each protein source with concentrations of 2.5%, 5.0%, and 10.0% (*w*/*v*) at a pH of 7.0. The pH effect was evaluated in 5.0% (*w*/*v*) concentrated protein solutions adjusted to pH of 5.0, 7.0, and 9.0 with 0.1 M NaOH or HCl solutions. 

The effect of Ca^2+^ ions was evaluated in concentrated protein solutions at 5.0% (*w*/*v*) and pH 7.0, incorporating CaCl_2_ at 1.0% (*w*/*v*) as the source of Ca^2+^ ions. The temperature effect was evaluated in all solutions. 

#### 2.4.1. Flow Behavior

Flow curves were obtained according to the methodology described by Zhu et al. (2018) with some modifications. Approximately 20 mL of each sample was taken, and the shear stress (τ) was measured as a function of the shear rate in three phases at 25 °C: an ascending curve (0.01–100 s^−1^ for 60 s), holding time (100 s^−1^ for 60 s), and a descending curve (100–0.01 s^−1^ for 60 s) [21]. Data from the descending curve were fitted to Herschel Hulkley’s model (best fit), and the consistency index (K) and flow behavior (n) for each protein solution were estimated.

#### 2.4.2. Oscillatory Measurements (Frequency Sweep)

The frequency sweep was assessed on solutions at 2.5% (*w*/*v*) and pH 7.0. The linear viscoelastic region (LVR) was evaluated at a constant angular frequency of 1 rad/s and a shear strain ranging from 0.001% to 100% [22]. Once the LVR was identified for each protein type, frequency sweeps were performed by varying the angular frequency from 10 to 0.1 rad/s at a constant temperature of 25 °C. This test measures the storage module (G’) along the phase shift angle (δ) [23].

#### 2.4.3. Temperature Sweep

The temperature sweep was used to determine the rheological parameter of proteins after thermal treatment. In order to induce gel formation, samples were subjected to a heating ramp composed of three steps: (i) from 25 °C to 80 °C (heating), (ii) 80 °C for 5 min, and (iii) from 80 °C to 25 °C (cooling). The heating and cooling rates were both 1 °C/min [24].

### 2.5. Statistical Analysis

Statistical analysis was performed using the Statgraphics^®^ software centurion version XVI (Statgraphics Technologies, Inc., The plains, Virginia, United States). The multi-way analysis of variance (ANOVA) test was used for data analysis, and *p*-values < 0.05 were considered significant. 

## 3. Results

### 3.1. Preliminary Studies 

Preliminary studies from the same authors evaluated relevant parameters such as free thiol group (SH) content, molecular weight (Mw), isoelectric point (pI), and amino acid composition (data unpublished). Those physicochemical aspects must be considered to understand the rheological behavior of the proteins being studied (Table 1) [16].

### 3.2. Influence of Protein Concentration and Thermal Treatment on the Rheological Properties

**Figure 1:**
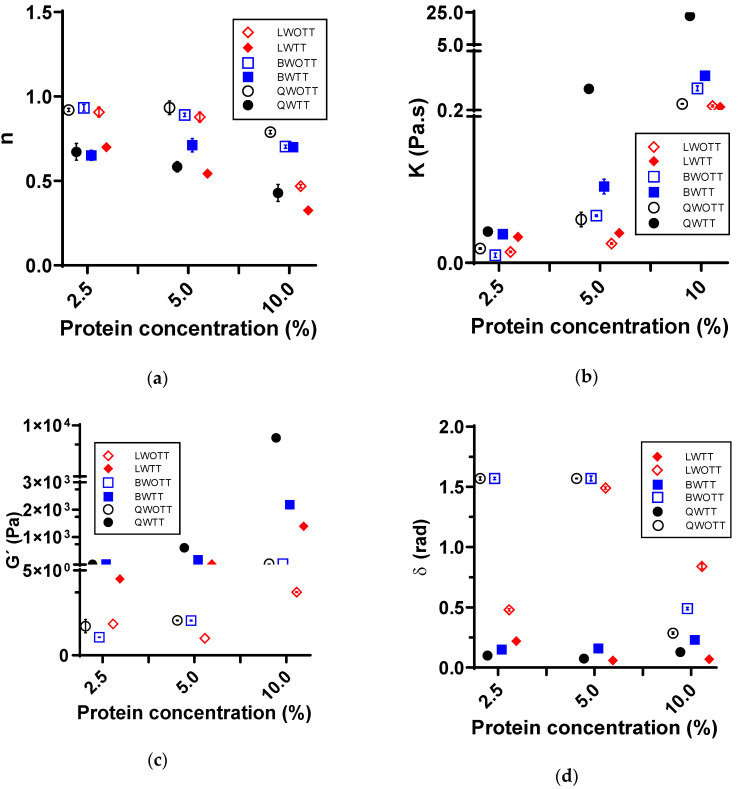
Effect of protein concentration and heat treatment on the rheological properties of solutions prepared with quinoa protein concentrates with heat treatment (QWTT) and without heat treatment (QWOTT); black bean protein concentrates with heat treatment (BWTT) and without heat treatment (BWOTT); and lentil protein concentrates with heat treatment (LWTT) and without heat treatment (LWOTT). (**a**) Average flow behavior (*n*); (**b**) average consistency index (K); (**c**) average storage module (G’) of the frequency sweeps; (**d**) average phase shift angle (δ) of the frequency sweeps.

Flow curves, frequency sweeps, and temperature sweeps were performed to determine the rheological properties of the extracted proteins from quinoa, black beans, and lentils. Flow curves and frequency sweeps were performed before and after each heat treatment (80 °C for 5 min) to identify rheological changes. Figure 1 shows the results obtained for parameters such as flow behavior (n), consistency index (K), storage module (G’), and phase shift angle (δ) for protein solutions at different concentrations of the three plant sources.

The flow behavior index (n) obtained for protein solutions (Figure 1a) reported a shear thinning behavior or pseudoplastic behavior (*n* ˂ 1) for all solutions independent of protein source, evaluated concentration, or heat treatment. However, when the protein concentration increased and without heat treatment, the n value decreased, and the protein solutions’ shear-thinning behavior increased. Generally, the heat treatment applied (WTT) decreased the *n* value compared to the treatment without heat (WOTT). The exception to this behavior was in the black bean protein solution (10%), where the *n* value obtained WOTT and WTT was similar. This implies that the effect of the concentration on the bean proteins’ flow behavior was more significant than the temperature’s impact compared to the other two protein sources. 

The effect of protein concentration and heat treatment on the consistency index (K) of protein solutions (Figure 1b) supports the impact generated on *n* values. The K value incremented for each solution when the protein concentration increased in both treatments WOTT and WTT. The K value expresses the necessary effort to make the solution flow; therefore, with a higher K, the solution is more viscous or elastic, being a shear-thinning behavior fluid with more K value than a Newtonian fluid [25].

Figure 1c,d show the average results of the storage module (G’) and phase shift angle (δ) obtained in the frequency sweep for each protein solution at different concentrations from the three plant sources. G’ for solutions increased as the protein concentration increased. In turn, δ decreased as the protein concentration increased. δ quantifies the relation G”:G’, indicating the relationship between the materials’ dissipated and stored energy. As δ decreased, the behavior of the sample went from a diluted solution (δ > 1.2 rad) to a viscous liquid (1.2 > δ > 0.7 rad) to a gel-type structure (δ < 0.7 rad) [25]. Therefore, when the protein concentration increased, the solution was driven from a viscous-type solution to a gel-type structure; the lower the δ, the higher the shear resistance. When applying heat treatment to the protein solutions, regardless of the evaluated concentration and the source, both G’ and δ significantly changed, increasing for G’ and decreasing for δ.

A considerable increase in G’ in WTT solutions was found, and it was directly proportional to the concentration and protein source. This indicates a higher G’ for isolated proteins from quinoa, then black beans, and finally lentils (Figure 1c). However, δ presented a considerable decrease in WTT solutions to a higher protein concentration. The protein source determined by the lowest δ value obtained was lentil proteins, followed by quinoa and, finally, black beans (Figure 1d).

### 3.3. Effect of pH on the Rheological Properties

**Figure 2:**
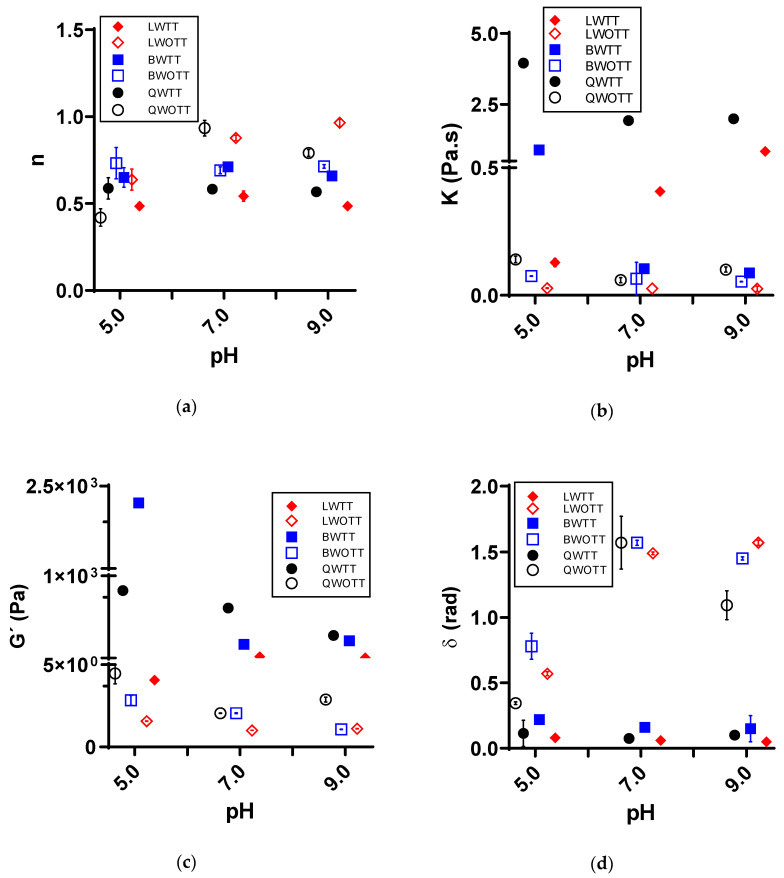
Effect of pH change and thermal treatment on the rheological properties of solutions prepared with quinoa protein concentrates with heat treatment (QWTT) and without heat treatment (QWOTT); black bean protein concentrates with heat treatment (BWTT) and without heat treatment (BWOTT); and lentil protein concentrates with heat treatment (LWTT) and without heat treatment (LWOTT). (**a**) Average flow behavior (*n*) of the solutions; (**b**) average consistency index (K) of solutions; (**c**) average storage module (G’) of the frequency sweeps; (**d**) average phase shift angle (δ) of the frequency sweeps.

Figure 2 shows the modifications in the rheological parameters of G’, K, n, and δ generated by the change in the medium’s pH, affecting the protein’s secondary structure. Figure 2a,b show the results obtained for n and K, respectively, for protein solutions at pH 5.0, 7.0, and 9.0. The *n* value continues to demonstrate a shear-thinning behavior of protein solutions. However, protein solutions at pH 5.0 have a higher shear-thinning behavior than at pH 9.0, where proteins have more solubility, increasing the *n* value and decreasing viscosity and interactions among molecules. The K values for the WOTT solutions were independent of the plant source and the pH; both remained constant. Nevertheless, the WTT solutions for quinoa and lentil proteins showed changes in both K and *n* parameters. The black bean protein solution with WTT at pH 5.0 presented a higher flow resistance than the other two pH-studied solutions. In contrast, the WTT lentil proteins showed an increase in K as the pH increased.

The parameters of G’ and δ (Figure 2c,d) presented differences between the solutions set to pH 5.0 compared with the solutions adjusted to pH 7.0 and 9.0, which reported G’ and δ values close to the WTT and WOTT solutions. The decrease in the adjusted pH to 5.0 generated an effect inversely proportional between G’ and δ. Figure 2c shows how, when decreasing the pH of the WOTT protein solutions, G’ had an increase regardless of the protein source. For the case of δ (Figure 2d), this parameter decreased when pH was adjusted to 5.0, strengthening the protein solution’s gel structure. These same WTT solutions at pH 5 reported δ lower than the WOTT solutions but comparable with the δ reported for the other protein solutions at pH 7.0 and 9.0 regardless of the plant source. On the other hand, G’ did show differences between the pH, the WTT and WOTT solutions, and the protein source, wherein WTT solutions at pH 5.0, module G’ was higher for the black bean, followed by the quinoa and then the lentil proteins. The gel formation behavior for the protein solutions from the three sources studied by varying their pH shows no significant difference between the G’s behaviors during the heat treatment of protein solutions from the same source to the three-pHs studied. However, the protein sources show differences, with the quinoa and black bean proteins having the highest G’ values. Nevertheless, as in the effect of protein concentration, δ minor was for those obtained using the protein isolated from lentils, without showing differences between the pH studied during the heat treatment.

### 3.4. Influence of Ca^2+^ on the Rheological Properties

**Figure 3:**
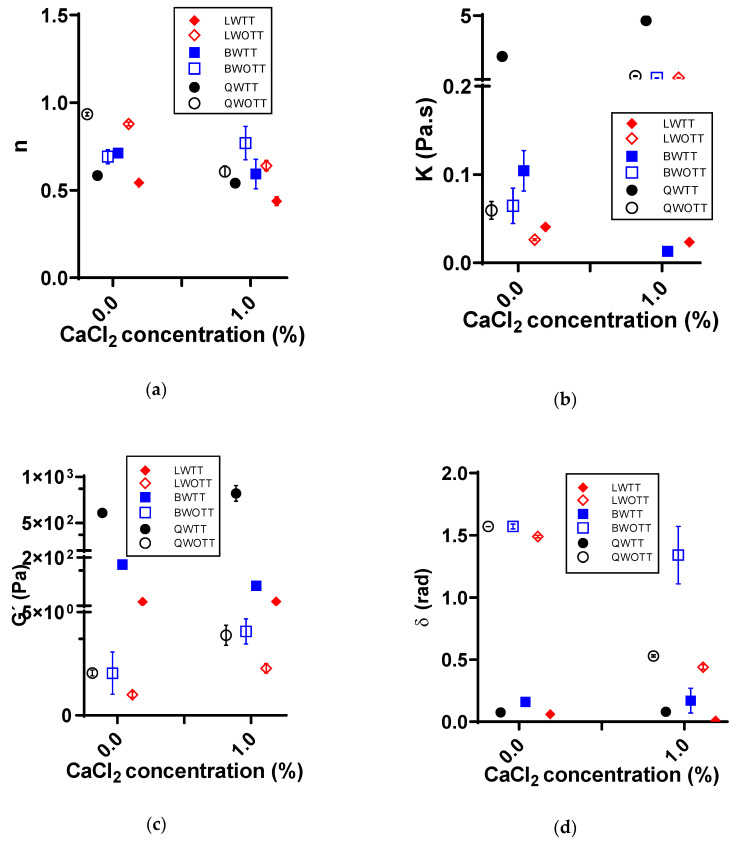
Effect of Ca^2+^ and thermal treatment in protein concentrates on the rheological properties of solutions prepared with quinoa protein concentrates with heat treatment (QWTT) and without heat treatment (QWOTT); black bean protein concentrates with heat treatment (BWTT) and without heat treatment (BWOTT); and lentil protein concentrates with heat treatment (LWTT) and without heat treatment (LWOTT). (**a**) Average flow behavior (*n*) of the solutions; (**b**) average consistency index (K) of solutions; (**c**) average storage module (G’) of the frequency sweeps; (**d**) average phase shift angle (δ) of the frequency sweeps.

The addition of CaCl_2_ to the WOTT protein solutions increased G’ and K and decreased n and δ. However, the WTT generated the proteins’ denaturation and the alignment of the rheological parameters of the solutions with and without CaCl_2_ (Figure 3).

The addition of salts to protein solutions led to a decrease in electrostatic repulsion forces, generating a rearrangement among proteins, increasing the crosslinking density between the polypeptide chains, and thus changing the rheological parameters of the solution. This behavior is described by the Derjaguin, Landau, Verwey, and Overbeek theory (DLVO) [26].

### 3.5. Statistical Analysis

The ANOVA was used to evaluate the effect of concentration, pH, Ca^2+^, and source type on the rheological parameters, such as consistency index (K), flow behavior (n), storage module (G’), and shift phase angle (δ). These results are shown below in Table 2.

## 4. Discussion

This work evaluated the variation effect of the concentration, pH, heat treatment, and presence of ions in protein solutions from three plant sources on the rheological properties, such as K, n, G’, and δ. The variations of the rheological parameters related to protein concentration in the WOTT solutions are attributed to electrostatic interactions and the molecular entanglement between proteins. The increase in the macromolecules’ density in the solution favored the electrostatic interactions and the molecular entanglement [21]. These interactions increased the resistance to shear or movement of the solution, expressed in the increase in viscosity, G’, and K, and decreased the n and δ values. These results are consistent with those reported by Chu L. et al. (2019) and Mu et al. (2019), which established that by increasing the number of soy protein molecules in a solution, macromolecules are close enough to become entangled, increasing its stable elastic behavior [21,25]. This behavior was inconsistent in the 10.0% lentil protein solutions without heat treatment; this lentil protein solution showed G’ values close to concentrated samples to 5.0%. This phenomenon could be attributed to the low molecular weight of lentil proteins (Table 1) compared to the other two plant sources studied. Therefore, only an increase in protein concentration in the solution will cause further macromolecular cross-linking. 

Heat treatment was a factor that significantly changed the rheological parameters studied, regardless of the source or concentration evaluated, increasing the elastic behavior of all protein solutions. This behavior can be attributed to the structural changes in the proteins. When the system’s temperature is increased, it promotes the unfolding of the protein’s secondary structure and exposes its reactive groups (–SH, COOH–, and −NH^3+^), generating other protein–protein interactions, possibly such as disulfide bridges, hydrogen bonds, or hydrophobic interactions [21]. Those interactions are responsible for the dominant elastic behavior rather than the viscous behavior during the gelation process. Proteins from lentils and black beans at a concentration of 10% obtained a lower G’ value than quinoa proteins at the same concentration after heat treatment. However, the δ that best expresses the solution’s behavior due to the relationship between G’ and G’’ showed that the gels obtained using the lentil proteins achieved a more defined and rigid structure than quinoa and bean proteins. This behavior can be attributed to the higher content of reactive amino acids (Cis, Arg, His, Lys, Asp, and Glu) in lentil proteins compared to the other two plant proteins (Table 1). Alternatively, the G’ and δ values for frequency sweeps showed increased values as the angular frequency for most protein solutions with WTT. These responses were independent of the concentration and protein source, demonstrating a frequency-dependent behavior and presenting gels with a weak structure.

The incorporation of H+ and OH− ions into the protein’s surrounding environment generated effects on the reactive moieties’ net charge and changed the electrostatic interactions that held the native protein structure. At pH 5.0, the incoming H+ ions induced a neutralization of acid groups, reaching the isoelectric points from quinoa and lentil proteins (Table 1). As a result, aggregates were formed, and the G’ and viscosity increased, whereas the δ decreased (Figure 2). Conversely, the surrounding OH− ions at pH 9.0 induced the ionization of carboxyl and thiol moieties, generating new covalent bonds and interactions with the medium, favoring globular proteins’ solubility as reported by Li et al. (2019) [27]. Further, there were no significant differences among the samples at pH 7.0 and pH 9.0. Once protein solutions suffered thermal treatment at different pH levels, peptide chains became mobile and hydrophobic, and reactive moieties were more accessible independent of the pH. As a result, the gel’s texture or viscosity were preserved with only small consistency differences.

Further, at pH 9.0, the net ionization charge of the moieties favored the formation of covalent bonds, strengthening the three-dimensional structure compared to soy and lentil proteins at pH 5.0, in which the net charge was close to zero. Puppo and Añón (1999) assessed the effect of pH on soy protein solutions on their viscosity behavior and found that the globular proteins (7 s and 11 s) increased the viscosity at a pH close to the proteins’ isoelectric point [28]. This behavior was comparable to those obtained in this study.

Proteins’ gelling properties are established on their ability to form three-dimensional networks. This depends upon the tertiary structure change of the proteins, either by a partial denaturation caused by thermal treatments or a change in their structure by the peptide links breaking [29]. The presence of Ca^2+^ ions during the protein gels’ formation increased coordinated ionic interactions between free carboxyl moieties present in the protein structure (amino acids such as Asp and Glu) and Ca^2+^ ions, strengthening the gel structure [26]. Results indicate that the elastic component of the solutions (G’) (Figure 3) increased without any thermal treatment for the three plant sources’ protein solutions. This change was possibly generated by the decrease in electrostatic repulsion between proteins by incorporating the Ca^2+^ ions, producing changes in the distribution and structure of proteins. These changes led to the crossbreeding and generation of new interactions that were reflected in the rheological parameters. However, upon thermal treatment, there was a great rheological change, forming a gel-like structure.

Results reported and analyzed in Figure 1, Figure 2 and Figure 3 are supported by statistical analysis (Table 2), indicating that the thermal treatment and protein concentration were the variables that generated a large number of significant effects (*p* < 0.05) on the rheological properties. Conversely, pH changes did not cause any significant alterations for any parameter, and Ca^2+^ only generated a substantial effect for the protein gels, which was directly associated with the gel network formation. Moreover, Table 2 shows the statistically significant effect (*p* < 0.05) of some covariates on the studied rheological parameters, indicating that variable δ contributes to n’s variability, and vice versa, and G’ contributes to K’s variability, and vice versa.

These results highlight the versatility of using vegetable proteins and how the conditions of the environment influence the rheological properties that can contribute to the design and development of food. Future research should focus on the behavior of these proteins in the incorporation of formulations of complex food matrices, such as meat derivatives, emulsions, and dairy products, among others, where there will be other factors, such as protein–starch, that can interact with proteins and potentiate or mask effects on techno-functional properties from proteins.

## 5. Conclusions

The incorporation of Ca^2+^, the increase in protein levels, and the decrease in pH close to the isoelectric point increased the solutions’ elastic behavior regardless of the protein source. Thermal treatment and protein concentrations caused the most significant impact on proteins’ rheological behavior, forming gels at levels higher than 5.0% protein, regardless of the source, pH, or Ca^2+^ ions.

## Figures and Tables

**Table 1:** Characterization of protein isolates obtained from quinoa, black beans, and lentils.

Amino Acid Composition (%)			
Amino Acids	Quinoa	Black Bean	Lentil
Cys	0.10	0.12	0.91
His	3.16	3.16	2.69
Arg	10.54	7.46	6.10
Lys	7.17	7.73	8.03
Asp	9.39	12.46	13.97
Glu	16.59	19.68	18.94
**Parameter**	**Quinoa**	**Black bean**	**Lentil**
MW (kDa)	58 and 46 to 32	80 to 32	46 to 32
pI	5.00 ± 1.00	3.50 ± 1.00	5.00 ± 1.00
SH (µM SH/g)	11.93 ± 0.45	19.16 ± 0.477	21.12 ± 0.27

Values are expressed as mean (*n* = 3); Mw: molecular weight, pI: isoelectric point, SH: thiol groups.

**Table 2:** ANOVA for the rheological parameters.

Parameters	n	k	G’	δ
	*p*-Value	*p*-Value	*p*-Value	*p*-Value
**Principal Effects**				
Source	0.4837	0.0075 *	0.1429	0.6301
Concentration (%)	0.8149	0.8370	0.0474 *	0.0019 *
pH	0.2686	0.6380	0.3361	0.2451
CaCl_2_ (%)	0.7601	0.7752	0.8926	0.0489 *
Thermal treatment	0.0023 *	0.0154 *	0.0013 *	<0.001 *
**Covariates**				
n	--------	0.1623	0.3102	<0.001 *
k	0.1623	--------	<0.001 *	0.2146
G’	0.3102	<0.001 *	--------	0.0813
δ	<0.001 *	0.2146	0.0813	--------

Significant differences (*p* < 0.05) according to LSD–Fisher (*). correlation analysis between parameters was not performed (--------). K: consistency index; *n*: flow behavior; G’: store module; δ: phase shift angle.

## Data Availability

Data available upon reasonable request.
